# Enhanced Detection of Viable *Escherichia coli* O157:H7 in Romaine Lettuce Wash Water Using On-Filter Propidium Monoazide-Quantitative PCR

**DOI:** 10.3390/microorganisms13010034

**Published:** 2024-12-27

**Authors:** Zhao Chen

**Affiliations:** 1 Joint Institute for Food Safety and Applied Nutrition, University of Maryland, College Park, MD 20742, USA; zhchen29@umd.edu; 2 Center for Food Safety and Security Systems, University of Maryland, College Park, MD 20742, USA

**Keywords:** *Escherichia coli* O157:H7, produce wash water, quantitative PCR, propidium monoazide, viability detection

## Abstract

Accurate detection of viable *Escherichia coli* O157:H7 in fresh produce wash water is critical for ensuring food safety and mitigating foodborne illnesses. This study evaluated an optimized on-filter propidium monoazide (PMA)-quantitative PCR (qPCR) method for detecting viable *E. coli* O157:H7 in romaine lettuce wash water, involving PMA pretreatment on a filter to block DNA amplification from dead cells. The method consistently detected viable cells across chemical oxygen demand (COD) levels of 1000 and 200 mg O_2_/L, with no significant differences (*p* > 0.05), indicating its tolerance to organic matter interference. Optimization experiments identified 10 µM PMA with a 10 min exposure time as the most effective pretreatment, achieving efficient inhibition of DNA from dead cells while preserving viable cell integrity. The limit of detection (LOD) was 1.3 CFU/mL, confirming its suitability for detecting low bacterial loads. Performance evaluations revealed that PMA-qPCR was accurate at viable-to-dead cell ratios of 1:10 or higher but became less reliable when dead cells outnumbered viable cells by a factor of 10 or more. The study demonstrates the potential of on-filter PMA-qPCR for routine food safety monitoring protocols in the fresh produce industry, while highlighting the critical role of viable-to-dead cell ratios in ensuring accurate detection, particularly in challenging samples with high dead cell loads.

## 1. Introduction

*Escherichia coli* O157:H7 is a leading cause of foodborne illness globally, with outbreaks frequently associated with fresh produce, particularly leafy greens such as romaine lettuce [1]. Produce wash water, a byproduct of postharvest processing, is a critical point in ensuring food safety, as it often serves as a reservoir for microbial contaminants, including *E. coli* O157:H7 [2]. During the washing process, microorganisms on the surface of fresh produce can transfer into the wash water, where they may persist or even multiply if not effectively managed [3]. Contaminated wash water poses a significant risk by facilitating cross-contamination, thereby contributing to the spread of foodborne pathogens throughout the supply chain [4]. Consequently, robust monitoring of wash water is essential for mitigating contamination risks and ensuring the microbial safety of fresh produce.

Traditional culture-based methods for detecting viable *E. coli* O157:H7 are labor-intensive, time-consuming, and less practical for rapid decision making during food processing [5]. Quantitative PCR (qPCR) has emerged as a highly sensitive and specific molecular tool for pathogen detection [6,7]. However, a major limitation of qPCR is its inability to distinguish between DNA from viable and dead cells, as it amplifies genetic material indiscriminately from both [8]. This shortcoming can lead to false-positive results, either prompting unnecessary corrective actions or creating an inaccurate assessment of contamination risk [9]. Moreover, organic matter and microbial debris in complex matrices such as produce wash water may interfere with DNA amplification [10]. Therefore, there is an urgent need for qPCR-based methods that can rapidly and accurately discriminate between viable and dead cells in high-risk environments like produce wash water.

To address this limitation, propidium monoazide (PMA) has emerged as a promising tool for enhancing qPCR specificity. PMA is a membrane-impermeable dye that selectively penetrates dead cells [11]. Upon exposure to light, PMA covalently binds to DNA, effectively preventing its amplification during qPCR. This selective inhibition ensures that only DNA from viable cells is amplified, offering a more precise assessment of microbial contamination. Despite its potential, successful implementation of PMA-qPCR in complex matrices like produce wash water requires careful optimization. The organic matter in produce wash water, derived from plant residues and soil particles, can hinder PMA penetration and qPCR amplification [10]. This highlights the need for tailored approaches to address such challenges effectively. Additionally, critical parameters such as PMA concentration and exposure time must be optimized to achieve accurate differentiation [12,13]. Suboptimal conditions can result in incomplete inhibition of DNA from dead cells or cytotoxic effects on viable cells, undermining the reliability of PMA pretreatment [14].

One notable improvement in PMA-qPCR sensitivity and reliability on water samples involves applying PMA directly to bacterial cells retained on filtration membranes, a method known as on-filter PMA pretreatment. This approach eliminates the bacterial resuspension step, reducing the risk of cell loss and enhancing the efficiency of viable cell detection. Previous studies demonstrated that on-filter PMA pretreatment significantly enhanced PMA-qPCR signal reduction for *Legionella pneumophila* in environmental or tap water samples, particularly at low bacterial concentrations [15,16,17], highlighting its potential for accurate quantification of pathogens in water with minimal contamination. However, the efficacy of on-filter PMA pretreatment depends on optimizing its conditions to accommodate the unique challenges posed by produce wash water.

In this study, we developed an optimized on-filter PMA-qPCR protocol to enhance the detection of viable *E. coli* O157:H7 in romaine lettuce wash water. Given the high organic load of wash water, we systematically evaluated the effects of PMA concentration and exposure time on viable cell detection while minimizing potential cytotoxic effects. By refining the on-filter PMA pretreatment method, we sought to establish a rapid and reliable strategy for distinguishing viable cells from dead ones in this complex matrix.

## 2. Materials and Methods

**Figure 1:**
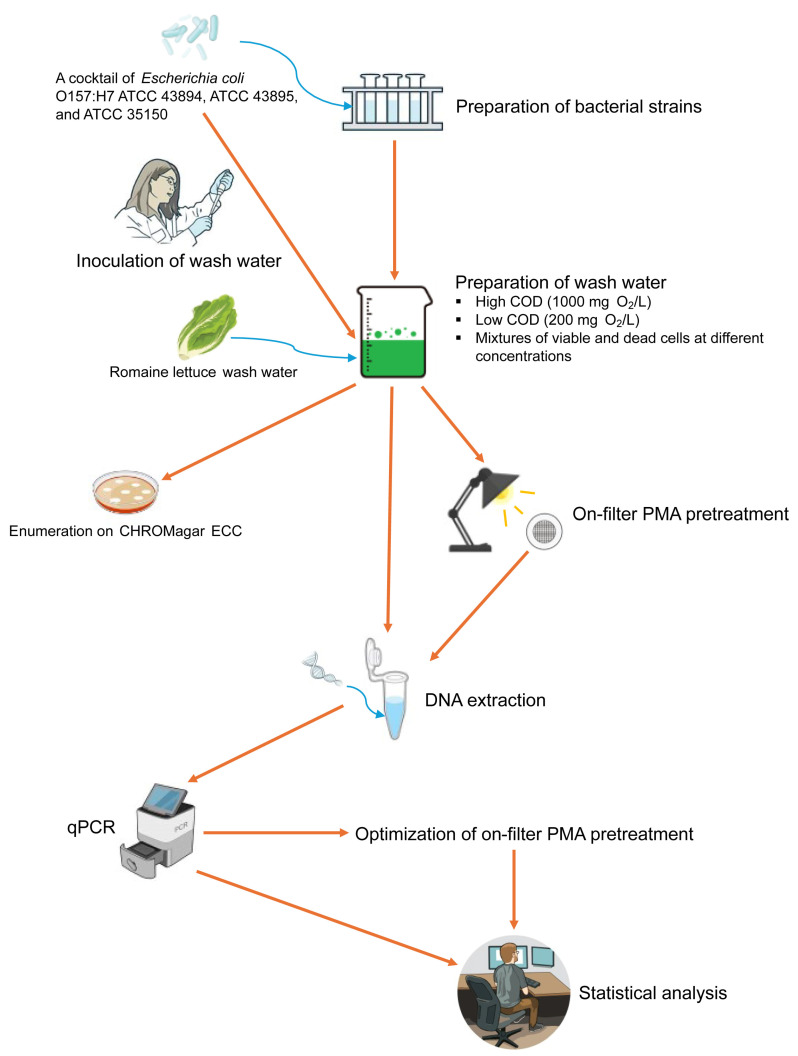
Flow chart depicting the experimental procedure for detecting viable *Escherichia coli* O157:H7 in romaine lettuce wash water using on-filter propidium monoazide (PMA)-quantitative PCR (qPCR).

A comprehensive flow chart illustrating the step-by-step experimental procedure employed in this study is presented in Figure 1.

### 2.1. Preparation of Bacterial Strains

A cocktail of three *E. coli* O157:H7 strains—ATCC 43894, ATCC 43895, and ATCC 35150—was used in this study. Stock cultures were maintained at −80 °C in tryptic soy broth (TSB; Fisher Scientific Inc., Hampton, NH, USA) supplemented with 25% glycerol. For preparation, each strain was streaked onto tryptic soy agar (TSA; Fisher Scientific Inc.) from frozen stock and incubated overnight at 35 °C. A single colony from each plate was transferred to TSB and subjected to two consecutive overnight incubations at 35 °C. Cells were harvested by centrifugation, washed with sterile Butterfield’s phosphate buffer (Fisher Scientific Inc.), and standardized to a concentration of 9 log CFU/mL (optical density at 600 nm: 0.7).

### 2.2. Preparation of Wash Water

Romaine lettuce (*Lactuca sativa* L. var. *longifolia*) was sourced from a local grocery store and confirmed to be free of *Escherichia coli* using the detection method specified by the U.S. Food and Drug Administration [18]. To prepare wash water, 50 g of lettuce was placed into a stomacher filter bag along with 100 mL of tap water and homogenized at 260 rpm for 5 min using a Seward Stomacher 400 Circulator (Seward Ltd., London, UK). The homogenate was diluted with tap water to produce wash water with a high COD level of 1000 mg O_2_/L, simulating lower-quality wash water often encountered during produce washing. The wash water was then diluted to achieve a lower COD level of 200 mg O_2_/L, resembling typical wash tank conditions as reported by Selma et al. [19].

COD levels were measured in triplicate using COD Digestion Vials, High Range (Hach Company, Loveland, CO, USA). After a 2 h digestion at 150 °C in a DRB200 Digital Reactor (Hach Company), COD levels were determined using the COD HR program on a DR900 Multiparameter Portable Colorimeter (Hach Method 8000, DOC316.53.01099; Hach Company). Both COD levels (1000 and 200 mg O_2_/L) were tested in subsequent experiments to evaluate the impact of varying organic matter concentrations on *E. coli* detection.

### 2.3. Inoculation of Wash Water

Viable cells were diluted in wash water to achieve final concentrations of 0, 1, 2, 3, 4, 5, 6, and 7 log CFU/mL. Dead cells were prepared by boiling bacterial suspensions at the same concentrations for 10 min to ensure complete cell death. Various mixtures of viable and dead cells were created by combining equal volumes (50 mL each) of viable and dead cells at different concentrations, as detailed in Table 1. Decimal serial dilutions of each mixture were prepared using sterile Butterfield’s phosphate buffer (Fisher Scientific Inc.). Each dilution was plated in triplicate onto CHROMagar ECC (CHROMagar, Paris, France) and incubated at 35 °C for 24 h to determine viable cell counts.

### 2.4. On-Filter PMA Pretreatment

On-filter PMA pretreatment was performed with modifications based on the methodology described by Slimani et al. [15] and Bonetta et al. [16]. PMAxx Dye (20 mM; Biotium, CA, USA), an advanced formulation of PMA with enhanced performance in suppressing PCR amplification of DNA from dead cells, was dissolved in distilled water to prepare a 10 mM stock solution, which was stored in darkness at −20 °C. Each 100 mL water sample was filtered through a 0.45 µm NEO-GRID membrane filtration system (Neogen Corporation, Lansing, MI, USA). The filter was overlaid with 1 mL of PMA, followed by a 15 min incubation in darkness at room temperature to ensure adequate PMA penetration. After incubation, the filter was placed on ice and exposed to a 500 W halogen lamp, positioned approximately 20 cm away, to induce photo-crosslinking.

### 2.5. DNA Extraction

Following light treatment, to avoid the bacterial resuspension step, DNA was extracted directly from the cells retained on the filter using an Aquadien DNA Extraction and Purification Kit (Bio-Rad Laboratories, Inc., Hercules, CA, USA).

### 2.6. qPCR

Primers targeting the *uidA* gene, encoding β-glucuronidase, were used to detect *E. coli* O157:H7 [20]. The sequences for PCR amplification were as follows: forward primer 5′-CAGTCTGGATCGCGAAAACTG-3′ and reverse primer 5′-ACCAGACGTTGCCCACATAATT-3′. The probe, labeled with a fluorophore and a quencher, was 5′-TET-ATTGAGCAGCGTTGG-MGB/NPQ-3′ (Applied Biosystems Inc., Foster City, CA, USA). PCR was performed in triplicate in a 20 μL reaction volume containing 4 μL of DNA template, 4 μL of nuclease-free water, 0.5 μL of 10 μM forward primer, 0.5 μL of 10 μM reverse primer, 1 μL of 5 μM probe, and 10 μL of SsoAdvanced Universal Probes Supermix (Bio-Rad Laboratories, Inc.). Negative controls were prepared by replacing the DNA template with 4 μL of nuclease-free water. Thermal cycling conditions were as follows: initial denaturation at 95 °C for 60 s, followed by 40 cycles of 94 °C for 10 s (optics off) and 63 °C for 40 s (optics on). All PCR reactions were conducted using a 7300 Real-Time PCR System (Applied Biosystems Inc.). Reactions were considered presumptively positive if any of the three targets crossed the threshold value within 40 cycles. The limits of detection (LODs) for qPCR and PMA-qPCR were evaluated using viable cell concentrations ranging from 7 log CFU/mL to 0 log CFU/mL. Standard curves at both COD levels were generated using 10-fold serial dilutions of viable cells to establish a linear relationship between cycle threshold (*C*_t_) values and viable counts (log CFU/mL).

### 2.7. Optimization of On-Filter PMA Pretreatment

Optimal conditions for on-filter PMA pretreatment were determined by systematically optimizing PMA concentration and exposure time using the previously described on-filter PMA pretreatment and qPCR procedures. The goal was to effectively distinguish between viable and dead cells while minimizing any potential cytotoxic effects. To determine the optimal PMA concentration, the filters with 100 mL of 5 log CFU/mL viable or dead cells were treated with PMA at concentrations of 5, 10, 15, or 20 µM for a fixed exposure time of 10 min. For determining the optimal exposure time, 10 µM PMA was applied to 100 mL of 5 log CFU/mL viable or dead cells retained on the filter for varying durations of 5, 10, 15, or 20 min. Control groups, consisting of 100 mL of 5 log CFU/mL viable and dead cells without PMA pretreatment, were included for comparison. The results from these optimization experiments informed the final conditions for the on-filter PMA pretreatment applied throughout the study.

### 2.8. Statistical Analysis

All data were obtained from three independent trials. Student’s *t*-test was performed using the t.test function in R 4.4.1 to determine whether significant differences existed. Figure plotting was performed using the ggplot2 3.5.1 R package [21].

## 3. Results and Discussion

### 3.1. Consistency of On-Filter PMA-qPCR Performance Across Varying COD Levels in Detecting Viable E. coli O157:H7 in Romaine Lettuce Wash Water

The results obtained for both COD levels were consistent, with no significant differences observed in *E. coli* O157:H7 detection efficiency (*p* > 0.05) (Figures S1 and S2). To streamline the presentation, only the data for the 200 mg O_2_/L COD level are shown. Similarly, Elizaquível et al. [10] demonstrated that PMA-qPCR results were consistent across high (500 mg O_2_/L) and low (2000 mg O_2_/L) COD levels when using this method to evaluate ultrasonic inactivation of *E. coli* O157:H7 in iceberg lettuce (*Lactuca sativa* L.) wash water. This consistency suggests that the optimized PMA-qPCR protocol is robust and unaffected by variations in organic load within the range tested. Organic matter, represented by the COD, can potentially interfere with PMA penetration and qPCR amplification by binding to the dye or causing matrix effects [14]. However, the lack of significant differences across COD levels indicates that the on-filter PMA pretreatment and optimized qPCR conditions effectively minimized such interference. By presenting data for the 200 mg O_2_/L COD level only, this study emphasizes the performance of the protocol under conditions resembling typical lettuce wash tank quality. Although higher COD levels are less common in well-maintained wash systems, testing the 1000 mg O_2_/L COD level confirmed the reliability of the protocol even under suboptimal conditions, further supporting its applicability across varying scenarios in the fresh produce industry. This robustness ensures that the method can be adopted confidently in monitoring microbial contamination, regardless of minor variations in wash water quality.

### 3.2. Optimization of On-Filter PMA Pretreatment for Detecting Viable E. coli O157:H7 in Romaine Lettuce Wash Water

Optimization of the on-filter PMA pretreatment protocol is essential to maximize its efficacy. Key parameters influencing the success of PMA treatment include the concentration of PMA used and the duration of exposure required to induce photo-crosslinking. While insufficient PMA concentration or exposure time may result in incomplete inhibition of DNA from dead cells, excessive PMA exposure could lead to cytotoxic effects on viable cells, reducing the accuracy of the method [22,23]. Thus, determining the optimal conditions for PMA pretreatment is crucial for its successful application in viability detection.

**Figure 2:**
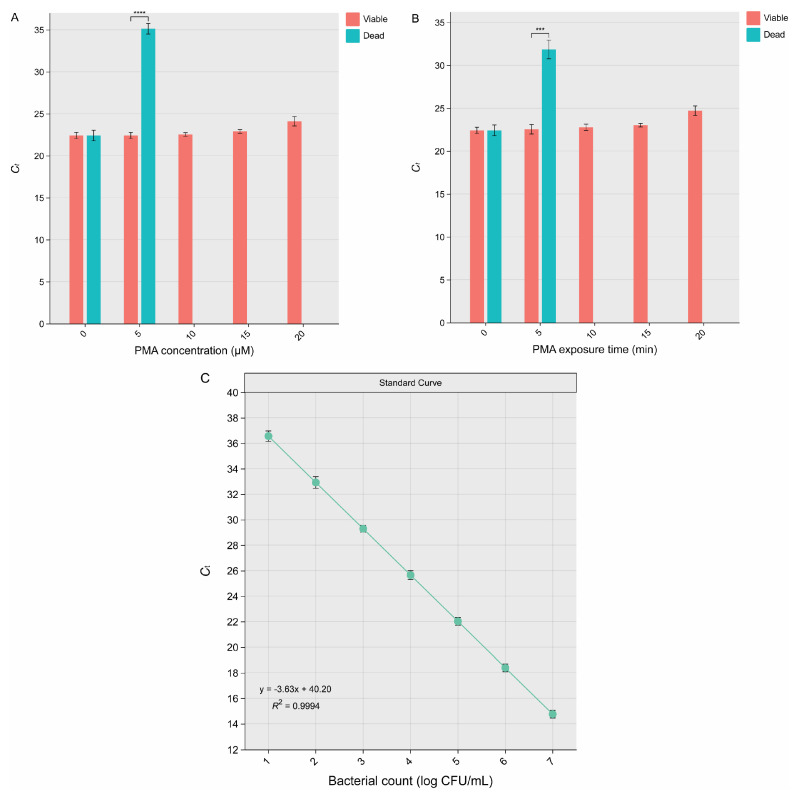
Optimization of propidium monoazide (PMA) concentration (**A**) and exposure time (**B**) of on-filter PMA-quantitative PCR (qPCR) for detecting viable *Escherichia coli* O157:H7 in romaine lettuce wash water at a chemical oxygen demand (COD) level of 200 mg O_2_/L using on-filter PMA-qPCR. Error bars represent the standard deviations from three independent trials. Asterisks are displayed to denote significant differences (***, *p* < 0.001; ****, *p* < 0.0001). Standard curve (**C**) of on-filter PMA-qPCR generated using 10-fold serial dilutions of viable cells to establish a linear relationship between cycle threshold (*C*_t_) values and viable counts (log CFU/mL). Error bars represent the standard deviations from three independent trials.

No dead cells were detected in wash water when treated with a PMA concentration of 10 µM (Figure 2A), indicating that this concentration was sufficient to inhibit amplification of DNA from dead cells. This finding establishes 10 µM as the minimal effective PMA concentration for distinguishing viable cells from dead ones. However, at a higher PMA concentration of 20 µM, cytotoxic effects on viable cells were observed, as evidenced by a significant increase in *C*_t_ (*p* < 0.05). These results suggest that excessive PMA concentrations may compromise the detection of viable cells due to unintended interactions with their intact cell membranes. The use of 10 µM PMA as the optimal concentration in subsequent experiments was a deliberate choice to balance effective inhibition of DNA amplification in dead cells while minimizing adverse effects on viable cells. This finding aligns with previous studies that reported a narrow range of effective PMA concentrations, often influenced by factors such as cell density, matrix composition, and sample type. Xie et al. [22] observed optimal PMA performance at 10 µM in inhibiting dead *E. coli* O157:H7 in irrigation water. In the study by Yuan et al. [23], the optimal PMA concentration varied with the season and turbidity of environmental waters: 10 µM PMA effectively inhibited DNA amplification from dead cells in winter samples, while 20 µM PMA was necessary for summer samples with higher turbidity, ensuring reliable differentiation between viable and dead *E. coli*.

An exposure time of up to 5 min did not effectively inhibit DNA amplification from dead cells (*p* < 0.0001), indicating that this duration was insufficient for complete PMA activation (Figure 2B). However, the inhibitory effect of PMA pretreatment improved progressively as exposure time increased from 0 to 10 min for dead cells. This trend suggests that longer exposure times enhance the ability of PMA to intercalate with the DNA of dead cells, thereby preventing their amplification during qPCR. Complete inhibition of DNA from dead cells was achieved with a 10 min PMA exposure, without negatively affecting viable cells. Conversely, extending the exposure time to 20 min resulted in cytotoxic effects on viable cells, as evidenced by reduced amplification, likely due to unintended interactions with intact cell membranes. Yuan et al. [23] identified a PMA exposure time of 10 min as optimal, effectively inhibiting DNA amplification from dead *E. coli* cells without affecting viable cells, while shorter times were insufficient and longer exposure (e.g., 20 min) risked reducing viable cell counts due to cytotoxic effects.

In this study, the optimal PMA pretreatment condition was a concentration of 10 µM PMA with a 10 min exposure time. This optimization underscores the critical need for carefully calibrating PMA concentrations and exposure times to suit specific experimental contexts. Overly aggressive PMA pretreatment can underestimate viable cell counts by inadvertently affecting intact cells, while insufficient treatment risks amplifying DNA from dead cells, leading to overestimations. The findings from this study validate the robustness of the optimized PMA pretreatment protocol, striking an effective balance between these extremes. This balance ensures reliable and accurate detection of viable *E. coli* O157:H7 in wash water, demonstrating its suitability under the tested experimental conditions.

### 3.3. Sensitivity of On-Filter PMA-qPCR for Detecting Viable E. coli O157:H7 in Romaine Lettuce Wash Water

Both qPCR and PMA-qPCR showed similar results when detecting viable cells in wash water (*p* > 0.05), demonstrating that both methods are equally effective in identifying low levels of viable cells. The theoretical LOD for the optimized PMA-qPCR method was determined using the standard curve equation y = −3.63x + 40.20 (Figure 2C), where y represents the *C*_t_ and x represents the concentration of viable cells (log CFU/mL). By setting the *C*_t_ threshold at 40, the corresponding x value was calculated as 0.1 log CFU/mL, indicating a theoretical LOD for PMA-qPCR of 1.3 CFU/mL. To further confirm this LOD, wash water at both COD levels was inoculated with viable cells at this concentration, and the cells were successfully detected using the PMA-qPCR method (*C*_t_ = 39.8).

While Xie et al. [22] and Yuan et al. [23] demonstrated robust detection in complex matrices, their sensitivity in environmental or irrigation water spiked with fecal *E. coli* or *E. coli* O157:H7 (1 and 2 log CFU/mL, respectively) underscores the challenges of working in turbid or organic-laden samples. In a study by Elizaquível et al. [10] evaluating PMA-qPCR for monitoring ultrasonic inactivation of *E. coli* O157:H7 in iceberg lettuce wash water, the reported LOD for the method was 1.3 log CFU/mL. In contrast, the optimized PMA-qPCR method achieved a LOD more than an order of magnitude lower, likely due to the efficient on-filter PMA pretreatment. This enhanced sensitivity makes it particularly suitable for applications requiring the detection of low contamination levels, such as stringent food safety protocols and routine monitoring in produce processing environments.

### 3.4. Impact of Viable-to-Dead Cell Ratio on PMA-qPCR Accuracy in Detecting Viable E. coli O157:H7 in Romaine Lettuce Wash Water

**Figure 3:**
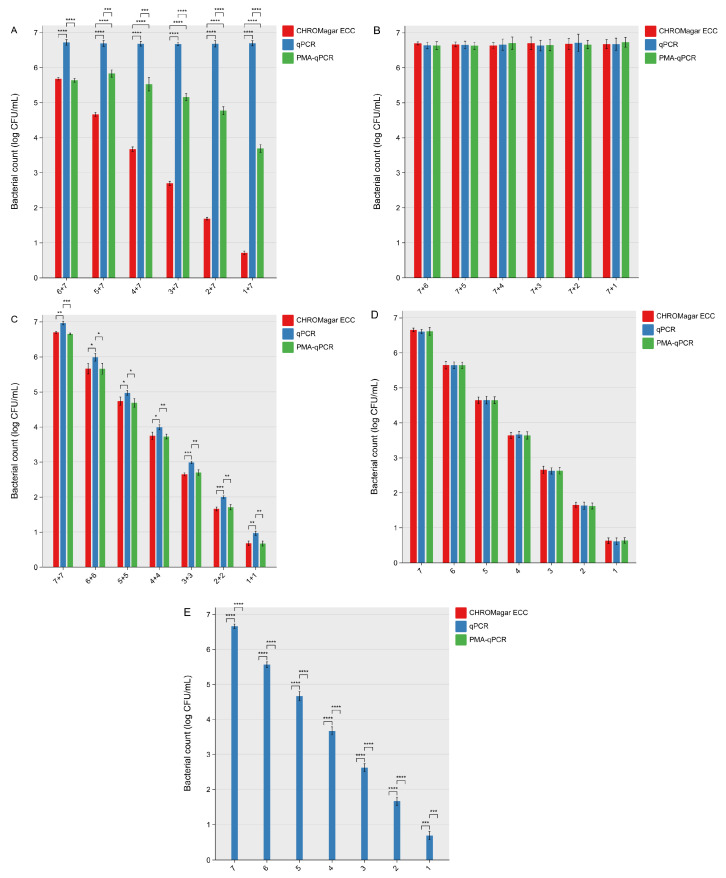
Use of propidium monoazide (PMA)-quantitative PCR (qPCR) to detect viable *Escherichia coli* O157:H7 in romaine lettuce wash water at a chemical oxygen demand (COD) level of 200 mg O_2_/L compared with CHROMagar ECC and qPCR: (**A**) Viable cell concentrations lower than dead cell concentrations; (**B**) Viable cell concentrations higher than dead cell concentrations; (**C**) Viable cell concentrations equal to dead cell concentrations; (**D**) Only viable cells present; (**E**) Only dead cells present. Error bars represent the standard deviations from three independent trials. Asterisks are displayed to denote significant differences (*, *p* < 0.05; **, *p* < 0.01; ***, *p* < 0.001; ****, *p* < 0.0001).

Viable or dead cells at 0 log CFU/mL were not detected using qPCR or PMA-qPCR. When the concentration of viable cells was lower than that of dead cells (Figure 3A), qPCR produced the highest bacterial counts (*p* < 0.001), reflecting its inability to distinguish between viable and dead cells. In contrast, PMA-qPCR provided accurate counts only when the viable-to-dead cell ratio was 1:10, yielding results similar to CHROMagar ECC (*p* > 0.05). However, when the concentration of dead cells exceeded that of viable cells by a factor of 10, PMA-qPCR became ineffective in differentiating viable cells from dead ones (*p* < 0.0001).

When viable cells were present at higher concentrations than dead cells (Figure 3B), all methods—CHROMagar ECC, qPCR, and PMA-qPCR—provided consistent bacterial counts, with no significant differences (*p* > 0.05). Similarly, when the viable-to-dead cell ratio was 1:1 (Figure 3C), CHROMagar ECC and PMA-qPCR delivered similar and accurate counts (*p* > 0.05), while qPCR again overestimated bacterial counts (*p* < 0.05) due to its inability to exclude DNA from dead cells.

When only viable cells were spiked into wash water (Figure 3D), all methods performed equally well, producing similar and accurate bacterial counts (*p* > 0.05). Conversely, when only dead cells were present (Figure 3E), CHROMagar ECC and PMA-qPCR detected no bacteria (*p* > 0.05), while qPCR produced false-positive results (*p* < 0.0001), emphasizing its limitation in distinguishing cell viability.

These findings align with previous studies, such as Kantonale Laboratorium [24], which demonstrated that the concentration of dead cells should not exceed that of viable cells by a factor of 100 for PMA-qPCR to function effectively. Similarly, Yang et al. (2011) found that the viable-to-dead cell ratio of *E. coli* must exceed 1:100 to ensure accurate detection [25]. Slimani et al. [15] reported that at viable-to-dead cell ratios of 1:10,000 or 1:100,000, the DNA-intercalating efficiency of PMA diminished significantly, resulting in the overestimation of viable cell counts when applied to *Legionella pneumophila* in saline. Yokomachi and Yaguchi [26] observed that for *E. coli*, the viable-to-dead cell ratio must exceed 1:10 to maintain a linear relationship between *C*_t_ values and viable cell concentrations. Yuan [27] further highlighted that high concentrations of dead *E. coli* cells in environmental waters reduced *C*_t_ values in mixtures, causing the overestimation of viable cells.

These results underscore the limitations of PMA-qPCR in samples where dead cells greatly outnumber viable cells. High concentrations of dead cells appear to reduce the availability of PMA for binding, compromising its ability to intercalate DNA effectively. Consequently, PMA-qPCR is better suited for monitoring samples with low bacterial loads rather than heavily contaminated samples dominated by dead cells. Future research should focus on enhancing the ability of PMA-qPCR to handle high loads of dead cells, potentially through improved PMA formulations, optimized pretreatment protocols, and alternative viability dyes. Additionally, exploring complementary techniques, such as integrating PMA-qPCR with advanced filtration or separation methods, could further refine its applicability in complex matrices with mixed bacterial populations. These advancements would expand the utility of PMA-qPCR for a broader range of food safety and environmental monitoring scenarios.

## 4. Conclusions

This study demonstrates the robustness and applicability of an optimized on-filter PMA-qPCR method for detecting viable *E. coli* O157:H7 in romaine lettuce wash water. The tolerance of this approach to varying organic loads and its high sensitivity makes it a reliable tool for low-bacterial-load applications, such as monitoring food safety in produce processing environments. This work contributes to improving detection strategies for foodborne pathogens, thereby advancing efforts to ensure food safety and public health. However, the accuracy of PMA-qPCR was influenced by the viable-to-dead cell ratio, with performance diminishing in samples where dead cells significantly outnumber viable cells. These findings underscore the importance of sample characterization and method calibration for optimal application. Future research should explore the application of on-filter PMA-qPCR for detecting a broader range of pathogens in diverse and complex matrices, including heavily contaminated samples. Emphasis should also be placed on optimizing advanced pretreatment protocols and integrating complementary techniques to overcome current limitations and enhance detection accuracy.

## Figures and Tables

**Table 1:** Compositions of viable and dead *Escherichia coli* O157:H7 cell mixtures in romaine lettuce wash water.

Group	Cell Concentration (log CFU/mL)	
	Viable Cells	Dead Cells
Viable cell concentrations lower than dead cell concentrations	6	7
	5	7
	4	7
	3	7
	2	7
	1	7
	0	7
Viable cell concentrations higher than dead cell concentrations	7	6
	7	5
	7	4
	7	3
	7	2
	7	1
	7	0
Viable cell concentrations equal to dead cell concentrations	7	7
	6	6
	5	5
	4	4
	3	3
	2	2
	1	1
	0	0
Only viable cells present	7	- ^a^
	6	-
	5	-
	4	-
	3	-
	2	-
	1	-
	0	-
Only dead cells present	-	7
	-	6
	-	5
	-	4
	-	3
	-	2
	-	1
	-	0

^a^ -, absence of viable or dead cells.

## Data Availability

The original contributions presented in the study are included in the article/Supplementary Material; further inquiries can be directed to the corresponding author.

## Supplementary Materials

The following supporting information can be downloaded at: https://www.mdpi.com/article/10.3390/microorganisms13010034/s1, Figure S1: Optimization of propidium monoazide (PMA) concentration and exposure time of on-filter PMA-quantitative PCR (qPCR) for detecting viable *Escherichia coli* O157:H7 in romaine lettuce wash water at a chemical oxygen demand (COD) level of 1000 mg O_2_/L using on-filter PMA-qPCR; Figure S2: Use of propidium monoazide (PMA)-quantitative PCR (qPCR) to detect viable *Escherichia coli* O157:H7 in romaine lettuce wash water at a chemical oxygen demand (COD) level of 1000 mg O_2_/L compared with CHROMagar ECC and qPCR.
