# Supplementary material for: Enhanced Detection of Viable Escherichia coli O157:H7 in Romaine Lettuce Wash Water Using On-Filter Propidium Monoazide-Quantitative PCR
Source: Microorganisms. 2024 Dec 27;13(1):34. doi: 10.3390/microorganisms13010034 (PMC11767674; doi:10.3390/microorganisms13010034)
Supplement: Supplementary file 1 [file microorganisms-13-00034-s001.zip › microorganisms-3392516-supplementary.pdf]

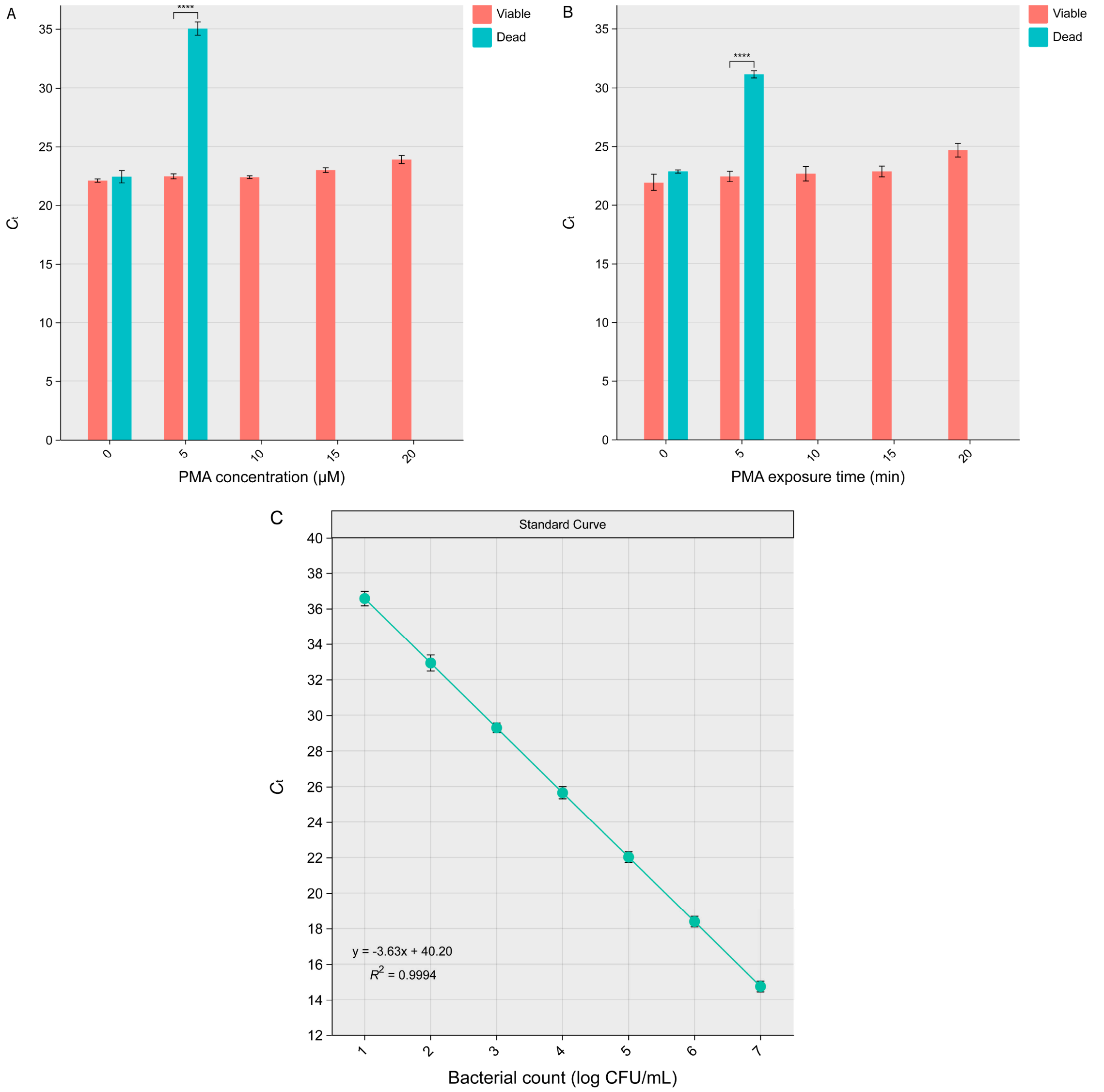

Figure S1. Optimization of propidium monoazide (PMA) concentration (**A**) and exposure time (**B**) of on-filter PMA-quantitative PCR (qPCR) for detecting viable *Escherichia coli* O157:H7 in romaine lettuce wash water at a chemical oxygen demand (COD) level of 1000 mg O_2/L using on-filter PMA-qPCR. Error bars represent the standard deviations from three independent trials. Asterisks are displayed to denote significant differences (****, $P < 0.0001$). Standard curve (C) of on-filter PMA-qPCR generated using 10-fold serial dilutions of viable cells to establish a linear relationship between cycle threshold (C_t) values and viable counts (log CFU/mL). Error bars represent the standard deviations from three independent trials.

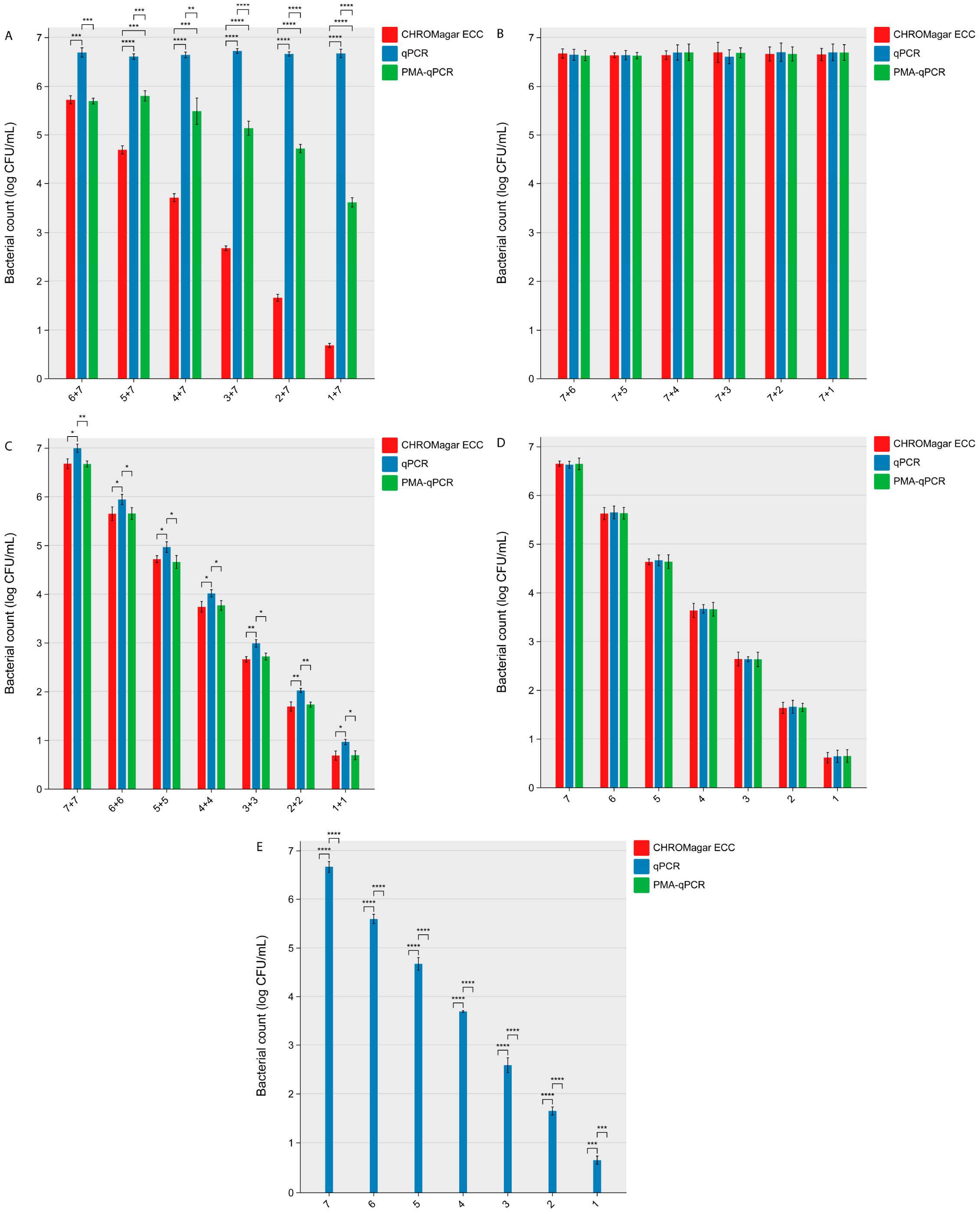

Figure S2. Use of propidium monoazide (PMA)-quantitative PCR (qPCR) to detect viable *Escherichia coli* O157:H7 in romaine lettuce wash water at a chemical oxygen demand (COD) level of 1,000 mg O_2/L compared with CHROMagar ECC and qPCR: (**A**) Viable cell concentrations lower than dead cell concentrations; (**B**) Viable cell concentrations higher than dead cell concentrations; (**C**) Viable cell concentrations equal to dead cell concentrations; (**D**) Only viable cells present; (**E**) Only dead cells present. Error bars represent the standard deviations from three independent trials. Asterisks are displayed to denote significant differences (*, $P < 0.05$; **, $P < 0.01$; ***, $P < 0.001$; ****, $P < 0.0001$).
